# Influence of Chloride Concentration on Fretting Wear Behavior of Inconel 600 Alloy

**DOI:** 10.3390/ma17122950

**Published:** 2024-06-17

**Authors:** Mengyang Zhang, Qinglei Jiang, Yizhou Zhang, Yinqiang Chen, Baoli Guo, Long Xin

**Affiliations:** 1 China Nuclear Power Operation Technology Corporation, Ltd., Wuhan 430223, China; zhangmy02@cnnp.com.cn (M.Z.); jiangql@cnnp.com.cn (Q.J.); zhangyz01@cnnp.com.cn (Y.Z.); chenyq02@cnnp.com.cn (Y.C.); 2 National Center for Materials Service Safety, University of Science and Technology Beijing, Beijing 100083, China; 18254987904@163.com

**Keywords:** steam generators, Inconel 600, fretting corrosion, Cl^−^ concentrations, fretting wear properties

## Abstract

The nickel-based alloy Inconel 600, strengthened by solution treatment, finds extensive application as a heat exchange pipe material in steam generators within nuclear power plants, owing to its exceptional resistance to high-temperature corrosion. However, fretting corrosion occurs at the contact points between the pipe and support frame due to gas–liquid flow, leading to wear damage. This study investigates the fretting wear behavior and damage mechanism of the nickel-based alloy Inconel 600 and 304 stainless steel friction pairs under point contact conditions in a water environment. Characterization was performed using laser confocal scanning microscopy and scanning electron microscopy equipped with energy-dispersive spectroscopy. Results indicate that the friction coefficient remains consistent across different chloride ion concentrations, while the wear volume increases with increasing chloride concentrations. Notably, friction coefficient oscillations are observed in the gross slip regime (GSR). Moreover, the stability of the oxide layer formed in water is compromised, diminishing its protective effect against wear. In the partial slip regime (PSR), friction coefficient oscillations are absent. An oxide layer forms within the wear scar, with significantly fewer cracks compared to those within the oxide layer in the GSR. It is worth noting that in GSR, the friction coefficient oscillates.

## 1. Introduction

Fretting wear is a prevalent occurrence in various dynamic systems and assembly contact components, with implications spanning diverse sectors such as aerospace [1], nuclear power generation [2,3], automotive manufacturing [4] and medical engineering [5]. Particularly within nuclear power plants, the steam generator, a pivotal component, faces heightened scrutiny due to its intricate material service environment [6,7]. In this context, the flow of water and steam within the secondary circuit not only induces vibration and fatigue at the contact points between pipes and supports where corrosion products accumulate, but also progressively thins the pipe walls, potentially leading to leaks and necessitating nuclear power plant shutdowns [8,9]. Consequently, in ensuring the safe operation of nuclear power plants, the impact of fretting wear demands meticulous attention [10,11,12].

Due to its exceptional high-temperature corrosion resistance, Inconel 600 alloy is commonly utilized as a material for heat exchange pipes in steam generators within nuclear power plants [13,14]. However, within the primary circuit system of nuclear power plants, a gap exists between the heat exchange pipeline of the steam generator and the support frame, leading to fretting wear at the contact points due to flow induced vibration [15]. Presently, numerous studies have delved into the fretting wear characteristics of Inconel 600 and Inconel 690 alloys. Lee and Kim [16] elucidated the effects of normal loading and displacement amplitude on fretting behavior between Inconel 600 MA and 690 TT alloys. Notably, they observed a lower wear rate of Inconel 690TT compared to Inconel 600 MA at room temperature. Jeong et al. [17] discovered that the friction coefficient and wear rate of Inconel 690 alloy was higher in air than in water. Furthermore, in investigating the influence of various displacement amplitudes on the fretting wear behavior of Inconel 600, Li et al. [18] observed an increase in wear volume and friction coefficient with displacement amplitude escalation, along with the presence of a nanocrystalline structure tribological transformed structure (TTS) during fretting wear. Lim et al. [19] researched the disparity between sliding wear and fretting wear of Inconel 690 and 600 in water at room temperature, concluding that the wear resistance of Inconel 600 surpassed that of Inconel 690 under fretting conditions. However, there have been limited direct evaluations on the effect of corrosion solution on the wear mode and microstructure of Inconel 600 alloy.

Studies have indicated the crucial role of constructing fretting maps in the investigation of fretting wear. These maps not only outline comprehensive behaviors, encompassing contact conditions, fretting regions, and damage mechanisms, but also delineate three fretting regimes: a partial slip regime (PSR), mixed fretting regime (MFR) and gross slip regime (GSR) [20,21,22]. In PSR, the tangential friction force linearly increases with displacement, forming a quasi-closed line. At the same time, slight slippage occurs at the contact edge without relative motion, with crack initiation at the interface. In MFR, the motion regulation mechanism is complex. Throughout various fretting processes, the contact surface may be dominated by either relative sliding adjustment, elastic deformation, or plastic deformation. In GSR, tangential friction exhibits significant fluctuations, particularly in the initial stages, yet the friction–displacement (Ft-D) curve typically forms a parallelogram, indicating noticeable relative slip between the two contacts [23,24]. 

In recent years, researchers have uncovered the significant corrosive effects of different ions present in water circuits on structural components. Zhang et al. [25] elucidated the impact of dissolved oxygen concentration in water on the fretting corrosion of Inconel 690 alloy, demonstrating that accelerated corrosion wear is a pivotal factor in fretting corrosion damage. Wang et al. [26] investigated the influence of pH value on the fretting wear behavior of Inconel 690 alloy. They observed that at a pH value of 1, the corrosion rate peaked due to the inability to form a passivation film on the surface. However, as the pH value shifted to 4, 7, and 10, a passivation film formed on the surface, significantly decelerating the corrosion rate. Park et al. [27] delved into the pit growth kinetics of Inconel alloy 600 in aqueous 0.1 M NaSO_4_ + 0.1 M NaCl solutions. Grégoire et al. [28] outlined the fundamental corrosion mechanisms of Inconel 600 alloy in molten chlorides. Despite the introduction of the corrosion mechanism of Inconel 600 alloy, scant attention has been directed towards the fretting wear of Inconel 600 alloy in a corrosive environment.

Therefore, this study aimed to investigate the impact of varying concentrations of corrosion solution on the fretting wear characteristics of Inconel 600 alloy. It further sought to delineate the fretting regime through the construction of friction force-versus-displacement amplitude curves, and to analyze the fretting morphology and microstructural alterations of the alloy in the corrosive solution so as to deeply understand its wear mechanism and oxidation behavior. Ultimately, the objective was to furnish a robust scientific foundation and guidance for the process design of pipe materials used in nuclear power steam generators.

## 2. Materials and Methods

The lower sample was a rolled and annealed Inconel 600 nickel-based alloy plate with a thickness of 2.1 mm. The chemical composition of the plate is shown in Table 1. The thin plate was cut into blocks measuring 15 mm × 15 mm × 2.1 mm, with the edges polished to ensure smoothness. Then, acetone was employed for ultrasonic cleaning to eliminate oil stains. Following cleaning, the test sample was affixed to a stainless base [29]. Subsequently, the surface underwent grinding using 2000# SiC metallographic sandpaper, succeeded by polishing with diamond polishing paste of 2.5 particle size until no discernible grinding marks remained (surface roughness Ra ≈ 0.02 μm).

The upper sample was a 304 stainless steel (SS) ball with a diameter of 10 mm. The material 304SS was a commonly employed anti-vibration component in CANDU nuclear power plants [30]. The chemical composition of the 304SS was detailed in Table 1.

The fretting wear tests were conducted using the SRV-IV fretting wear sample machine, featuring a ball-on-plate configuration, manufactured by Optimol Company in München, Germany. The structure of the fretting wear working area and the clamping device diagram for point contact can be referenced in prior studies [31,32]. This SRV fretting wear tester allowed the simultaneous recording of four parameters: the friction coefficient (FC), friction force (Ft), displacement amplitude (D), and normal load throughout the fretting wear test. Utilizing the FSA module configuration, 1024 data points can be recorded in each cycle. Data processing enabled the generation of Ft-D fretting diagrams during the test process. All experiments were conducted under point contact conditions.

In the investigation of fretting damage characteristics in nickel-based alloys influenced by Cl^−^, the friction medium comprised 0.9%, 3.5%, 5%, 8%, 12% NaCl solution, and deionized water. Experimental parameters selected for the gross slip regime (GSR) included a normal force of 100 N, a frequency of 20 Hz, and a displacement amplitude of 45 μm, with a test duration of 30 min. For the partial slip regime (PSR), experimental parameters were set at a normal force of 150 N, a frequency of 5 Hz, and a displacement amplitude of 30 μm, with a test duration of 40 min. To ensure result repeatability, three tests were performed under each condition.

After the wear test, the specimens underwent ultrasonic cleaning in acetone and alcohol for 10 min, respectively, followed by drying with compressed air. The wear volume and profiles were assessed using a laser scanning confocal microscope (LEXT OLS4000, Olympus, Tokyo, Japan). Scanning electron microscopy (SEM) equipped with an energy-dispersive spectroscopy (EDS) detector was employed to analyze the surface morphology of the wear scar, and acquisition of elemental composition data from various regions and specialized morphological features of the wear surface.

## 3. Results and Discussion

### 3.1. Running Condition Fretting Map in Water Environment

**Figure 1:**
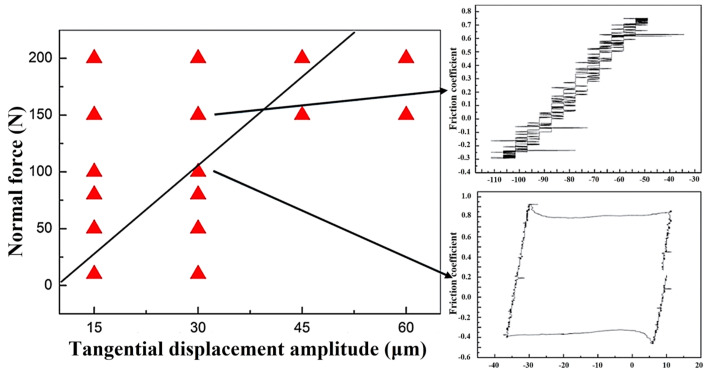
Running condition fretting map in deionized water conditions with the typical Ft-D curves.

As shown in Figure 1, under the condition of deionized water, multiple experiments were conducted to attain a stable state. Based on the data obtained from this stable state, Ft-D curves and a running condition fretting map (RCFM) were generated. It was evident from the RCFM that Inconel 600 alloy exhibits a combination of the partial slip regime (PSR) and gross slip regime (GSR), with the mixed fretting regime (MFR) being less prevalent. Specifically, when the displacement amplitude was small and the applied load was large, the PSR was predominant, whereas the GSR prevailed under contrasting conditions [1]. From Figure 1, with the decrease in normal load and the increase in displacement amplitude, the fretting regimes change from PSR to GSR. In PSR, the Ft-D curves are close to a quasi-closed line. In GSR, the Ft-D curves are gradually pulled apart, most of which keep a quasi-parallelogram shape. In the SRV experiments, the amplitude change in this region is obvious, resulting in different expansion of the Ft-D curve.

Zhou et al. [23] proposed that the mixed fretting regime (MFR) represented an unstable behavior arising from the evolution of the wear surface and the generation of loosened debris within the contact interface. However, MFR proved challenging to observe in the case of Inconel 600 alloy due to its narrow operational range. Further investigation and refinement of parameters are necessary to validate this phenomenon. It was widely recognized that establishing MFR in brittle materials was difficult [33]. Additionally, when water lubrication is introduced, the fretting regime of Inconel 600 alloy predominantly comprises a partial slip regime (PSR) and gross slip regime (GSR).

### 3.2. Effect of Fretting Wear Behavior in Different Cl^−^ Concentrations

#### 3.2.1. The Fretting Regimes in GSR

**Figure 2:**
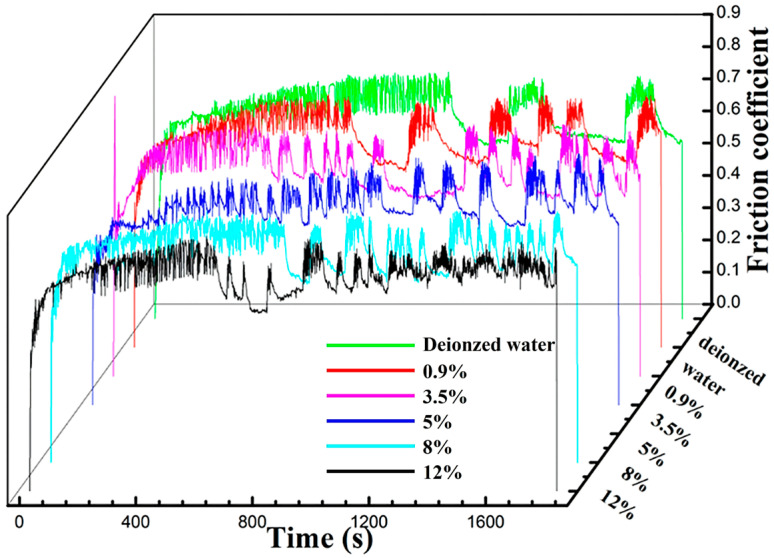
The variation curves of the friction coefficient with time in solutions of different Cl^−^ concentrations.

Figure 2 shows the variation curves of the friction coefficient over time at various Cl^−^ concentration solutions. As shown in Figure 2, in GSR, friction coefficient curves in different water media exhibit significant fluctuation characteristics, with evident seismic oscillations observed throughout the entire testing duration. Particularly noteworthy is the stability of the friction coefficient curve for the sample immersed in deionized water (green curve) before 1200 s, where no oscillations are observed. However, as the Cl^−^ concentration increases, oscillations emerge between 600 s and 1000 s. Generally, the friction coefficient oscillates between 0.55 and 0.75, with the highest recorded friction coefficient reaching approximately 0.7.

**Figure 3:**
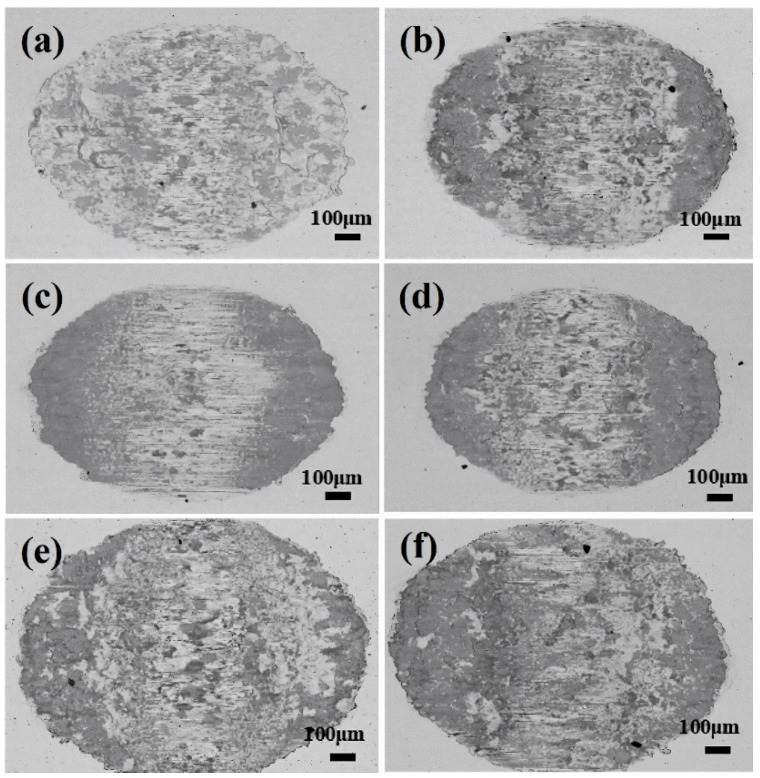
The surface SEM (BSE mode) morphologies of the wear scars in different Cl^−^ concentrations. (**a**) Deionized water, and (**b**) 0.9%, (**c**) 3.5%, (**d**) 5.0%, (**e**) 8.0%, and (**f**) 12.0%.

Figure 3 shows the surface morphologies of wear scars observed through SEM in BSE mode under various Cl^−^ concentration solutions, contrasting with dry friction conditions under identical fretting test parameters [18]. Based on the contrast between light and dark regions in the SEM images, it can be inferred that the darker regions represent the oxide layer while the brighter regions represent the matrix [34]. Specifically, it can be found from Figure 3a–f that the oxide film formed in the central contact area of the wear scar exhibits incompleteness, with localized shedding of the oxide film observed on both sides of the wear scar. The mechanism underlying the formation of incomplete oxide layers is multifaceted. On the one hand, the limited presence of dissolved oxygen in water constrains the rate and integrity of oxide layer formation. On the other hand, wear debris initially formed tends to diffuse into the solution during the frictional process following oxidation, thus hindering the formation of a dense oxide layer.

**Figure 4:**
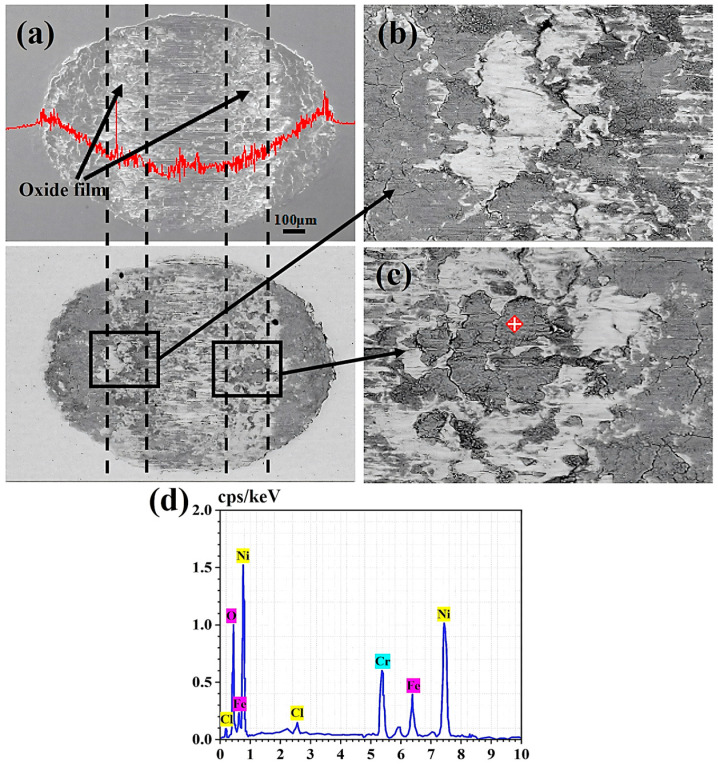
The surface characteristics and oxide energy spectrum analysis results after fretting wear in 0.9% Cl^−^ concentration solution. (**a**) Wear surface SEM morphologies and BSE morphologies, (**b**,**c**) the magnified SEM images of the rectangular regions in (**a**), and (**d**) the EDS results of marker points in (**c**).

Taking the fretting wear results in 0.9% Cl^−^ concentration solution as an example [35], the wear damage characteristics in a water environment were analyzed. Figure 4a shows the wear morphology of the sample and the wear profile traced along the center of the wear scar. Notably, the wear scar exhibits a semi-elliptical shape, while the BSE image reveals the emergence of a black oxide layer at the bottom edge of the wear scar. Figure 4b,c show the magnified morphology at the black rectangular position in Figure 4a. In Figure 4b, the oxide layer appears to peel off from the white substrate, with numerous cracks observed at the junction of the substrate and the oxide layer. Figure 4c further reveals the serious oxidation phenomenon at the crack. Figure 4d shows the energy spectrum results of the marker points in Figure 4c. It can be found that O and Cl were detected on the worn surface, indicating oxidation occurs during fretting wear in the case of 0.9% Cl^−^.

**Figure 5:**
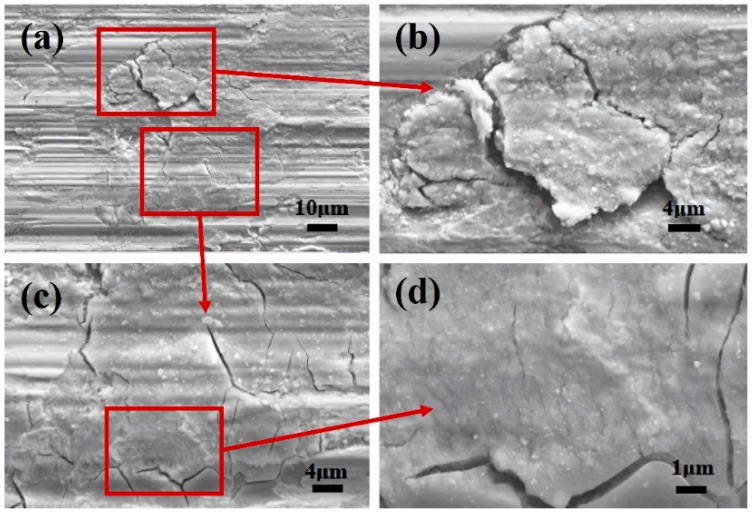
(**a**) The SEM morphology of worn scar center after fretting wear in 0.9% Cl^−^ concentration solution. (**b**) The magnified morphology of delamination. (**c**,**d**) The magnified morphologies of cracks.

Figure 5 shows the SEM morphologies of the wear scar center following fretting wear in a 0.9% Cl^−^ concentration solution. The prominent grooves aligned with the fretting direction can be observed in Figure 5a. Further magnification of the morphology reveals the presence of cracks along the fretting direction, as depicted in Figure 5c,d, along with nubbly delamination observed in Figure 5b.

Comparing the fretting results under dry friction conditions, a notable distinction emerged when fretting wear tests were conducted in a water medium: the difficulty in forming a third body layer observed under dry friction conditions [18]. This difficulty primarily arose from the tendency of oxides to disengage from the contact surface as fretting progresses in the aqueous environment, hindering the formation of a continuous and dense third body layer. As illustrated in Figure 4 and Figure 5, the absence of this third body layer in the water medium results in metal-to-metal contact in localized central areas, leading to the formation of micro-cracks on the surface. These micro-cracks not only constitute a structural defect but also serve as potential channels for oxygen diffusion, exacerbating surface oxidation, as noted in the literature [36]. Moreover, the differential microstructures between the oxide layer and the substrate render the oxide layer more prone to peeling. As depicted in Figure 5, this peeling action results in a rougher friction surface, further influencing frictional performance.

**Figure 6:**
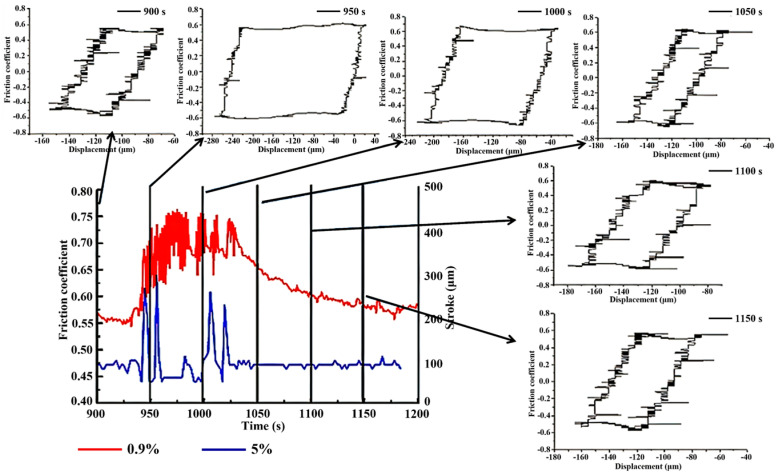
The variation curves of the friction coefficient with time and the evolution of Ft-D curves.

Figure 6 shows the variation curve of the friction coefficient under different Cl^−^ concentrations during the period from 900 s to 1200 s. The red curve represents the change in friction coefficient at a 0.9% Cl^−^ concentration, while the blue curve represents the change in friction coefficient at a 5% Cl^−^ concentration. As shown in Figure 6, it can be seen that the increase in the friction coefficient correlated with a sudden increase in amplitude, as analyzed through the change curve of the FSA module and the friction coefficient amplitude over time, as well as the variation in the Ft-D curve during the intermediate process. 

Significantly, when the amplitude experiences a sudden increase, the friction coefficient concurrently increases. However, as the amplitude stabilizes, the friction coefficient gradually decreases. This oscillatory phenomenon is attributed to the surface condition. During periods of stability, wear debris are discharged with the flow of water. However, during the central metal-to-metal contact state characterized by reciprocating motion, the region experiences significant contact tangential stress. Upon the detachment of the bulk oxide formed at the central contact position, stress is suddenly released, leading to a rapid expansion of displacement towards the oxide layer areas at both ends, as demonstrated in Figure 4. This process induces a sudden increase in the area enclosed by the hysteresis curve, resulting in surface roughening and a rapid increase in the friction coefficient. Subsequently, as the amplitude stabilizes, the friction coefficient decreases to a stable value.

**Figure 7:**
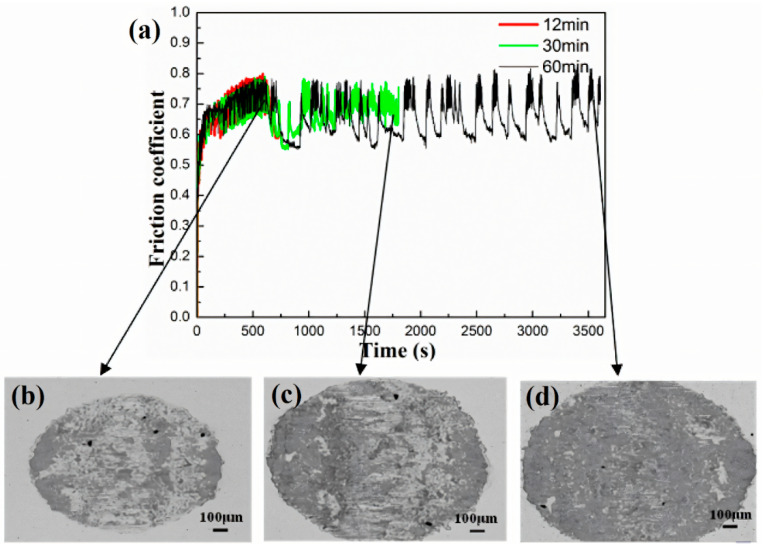
The variation curve of the friction coefficient with time (**a**) and the surface BSE morphology images of the wear scar (**b**–**d**) in 12% Cl^−^ concentration solution at different stopping times.

Figure 7 shows the variation curve of friction coefficient over time in a 12% Cl^−^ concentration solution, along with surface BSE morphology images captured at different intervals throughout the test. In these images, the darker regions correspond to the oxidized portions resulting from the test. Analysis of Figure 7b reveals that at the onset of the test, the wear surface exhibits non-uniformity, primarily comprising the bare matrix with distinct blocky oxide layers. As the fretting wear test proceeds, the width of the wear scar gradually increases, and the surface tends toward flatness, as shown in Figure 7c. Eventually, the entire wear scar area becomes enveloped by an oxide layer, as shown in Figure 7d. However, it is noteworthy that after 500 s, the friction coefficient continues to exhibit periodic fluctuations, failing to stabilize at a constant value. This phenomenon was attributed to the energy dissipation due to the variation of friction/wear periods [37,38]. This observation further underscores that the oxide layer formed in the aqueous environment lacks stability, consequently failing to effectively mitigate wear.

In the fretting wear test, the gradual breakdown of the original oxide layer due to continuous friction resulted in direct metal-to-metal contact. This direct contact significantly increased the actual contact area, leading to surface adhesion and plastic deformation acting simultaneously on the worn surface. Consequently, there was a rapid elevation in the friction coefficient of the worn surface [39]. As shown in Figure 2 and Figure 7, noticeable oscillations were observed in the curve of friction coefficient over time. These oscillations stemmed from the fretting test parameters and the conditions of the aqueous environment, where the normal force was insufficient and the displacement amplitude was excessive, resulting in the amplitude not capable of being stabilized at the initial displacement amplitude. Additionally, the oxide layer was prone to peeling off under the influence of water flow, promoting metal-to-metal contact between the friction pair. In this scenario, the localized oxide spalling diminished wear resistance, causing a sudden increase in amplitude, and consequent friction coefficient rise. Furthermore, the entire test process occurred in GSR, resulting in a low signal-to-noise ratio and inaccurate experimental outcomes. To analyze the influence of Cl^−^ on fretting damage behavior accurately, adjustments to the fretting parameters should be made based on the operating conditions fretting map. Specifically, the increase in the normal force and the decrease in the displacement amplitude would shift the fretting regime to PSR, as shown in Figure 1. This adjustment facilitates the study of fretting damage behavior under corrosive solutions.

#### 3.2.2. The Fretting Regimes in PSR

**Figure 8:**
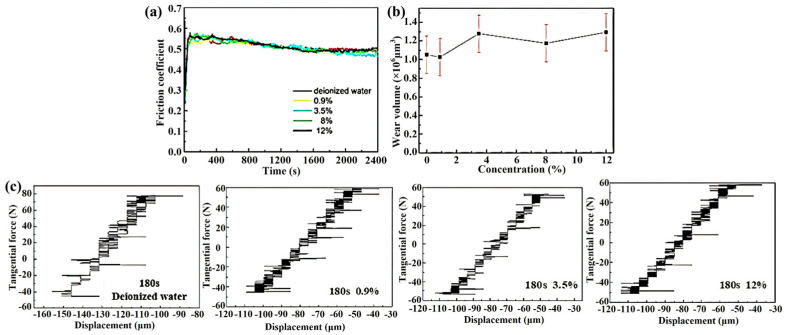
The fretting test results in solutions with different Cl^−^ concentrations. (**a**) The variation curves of FC with time, (**b**) the variation curve of wear volume with different Cl^−^ concentrations, and (**c**) the Ft-D curves under different Cl^−^ concentrations at 180 s.

Based on the previous conclusions regarding the instability of friction amplitude and the low signal-to-noise ratio associated with parameters in GSR, adjustments are made in accordance with the running condition fretting map to select parameters falling within the PSR. As shown in Figure 8a, under the adjusted experimental conditions, the friction coefficient curves exhibit stability and demonstrate characteristic behavior indicative of PSR. Notably, the friction coefficient initially reaches 0.57 and eventually stabilizes at approximately 0.5. In Figure 8b, the variation curve of wear volume is depicted. Within the margin of error, the wear volume consistently hovers around 1.1 × 10^6^ μm^3^, a significant reduction compared to the wear volume observed in the GSR. Furthermore, different concentrations of Cl^−^ solutions have minimal effect on the wear volume. Figure 8c shows the Ft-D curves of each sample under various Cl^−^ concentration solutions at 180 s. These curves validate that the fretting is indeed in the PSR.

Vingsbo and Söderberg first introduced the concept of fretting maps in their research [20], while Zhou further divided the fretting regimes into three distinct types: PSR, MFR and GSR [40]. In the current study, both PSR and GSR were observed in Figure 6 and Figure 8. However, MFR was not easy for the Inconel 600 alloy [41]. In GSR, as the amplitude increases, the FC shows a corresponding upward trend, as shown in Figure 6. At 950 s, the FC suddenly increased significantly, accompanied by a sudden increase in the area enclosed by the Ft-D curve. Subsequently, as the displacement amplitude gradually stabilized, the FC decreased gradually to a stable level. Relatively speaking, in PSR, the Ft-D curve maintained a quasi-closed line, as shown in Figure 8. At 180 s, varying concentrations of Cl^−^ corrosion solution did not significantly affect the fretting regime. However, with continuous fretting wear testing, a phenomenon of motion lag emerged, leading to the gradual transformation of the quasi-closed line into a shape resembling a quasi-parallelogram. 

By comparing the changes in Ft-D curves of the Inconel 600 alloy in GSR and PSR, it can be found that the water environment with different concentrations has a significant influence on the wear of the sample in GSR, while its impact in PSR is relatively minor. This phenomenon primarily stems from the formation and exfoliation of the oxide layer during the wear process. In GSR, an incomplete oxide layer is formed on the surface of the Inconel 600 alloy, as shown in Figure 5. These oxide layers served a lubricating function during the wear process. However, as the test progressed, the effect of water flow made the gradual peeling off of the oxide layer from the wear surface. Therefore, localized areas of direct metal-to-metal contact occurred on the contact surface, exacerbating wear and facilitating the formation of micro-cracks, which further accelerated oxide layer peeling. In PSR, the oxide layer formed on the worn surface was less prone to peeling off, allowing it to effectively reduce friction. Therefore, in PSR, varying concentrations of water environment have minimal effect on the wear of the sample.

**Figure 9:**
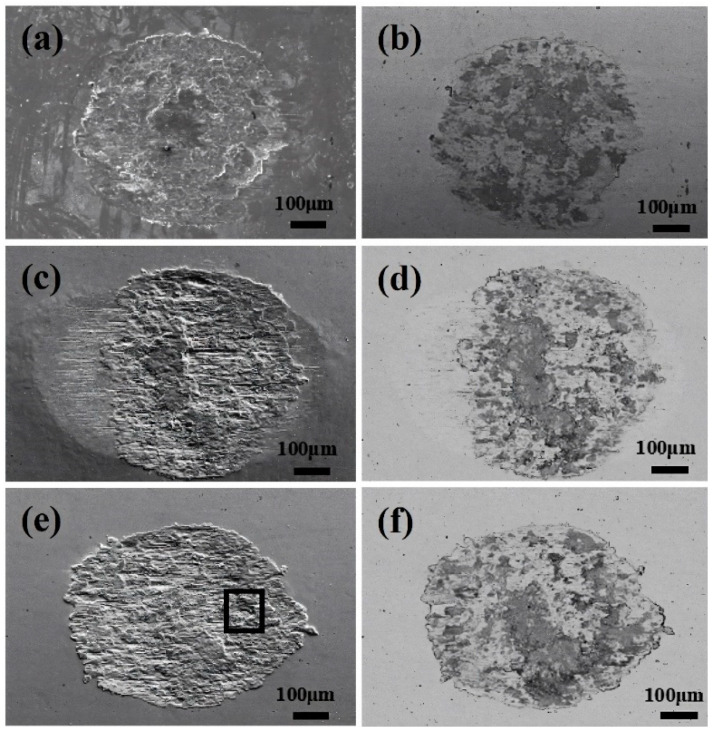
The surface SEM and BSE morphologies of the wear scars at solutions with different Cl^−^ concentrations. (**a**,**b**) Deionized water, (**c**,**d**) 3.5%, (**e**,**f**) 8.0%.

Figure 9 shows the morphology of the wear surface in PSR, with Figure 9a,b is the morphology under deionized water, Figure 9c,d is the morphology under 3.5% Cl^−^ concentration, and Figure 9e,f is the morphology under 8.0% Cl^−^ concentration. Unevenly distributed adhesion is observed on the worn surface, which indicated that this adhesion corresponds to an oxide layer. In deionized water, an oxide layer primarily is formed in the central region of the wear surface. With an increase in Cl^−^ concentration, the area covered by the oxide layer increases. Specifically, Cl^−^ was an aggressive ion that accelerated the oxidation process on the metal surfaces [42,43,44]. However, it is noteworthy that even with an increase in Cl^−^ concentration, the oxide layer remains primarily in the central region of the wear surface, as shown in Figure 9a,c,e. As the Cl^−^ concentration increases, cracks start to form, as shown in Figure 9b,d,f. This occurrence is primarily attributed to the continuous collision and extrusion of the upper sample, leading to the formation of cracks and oxidation at both ends of the material, ultimately resulting in delamination.

**Figure 10:**
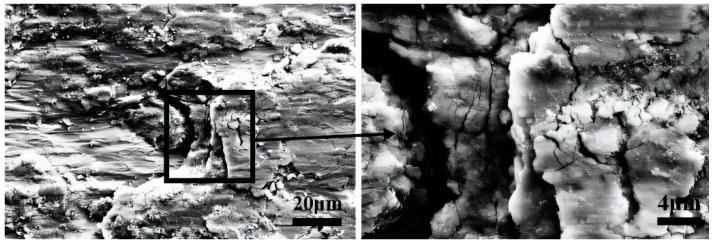
The detailed SEM morphologies of the wear scar center after fretting wear in 8.0% Cl^−^ concentration solution.

Figure 10 shows detailed SEM images of the rectangular area in Figure 9e. When the Cl^−^ concentration is 8.0%, a crack is formed. Figure 10 shows the evolution of crack formation and oxidation. The continuous impact and compression impose on both ends of the wear scar by the upper sample initiate cracks. These cracks not only compromise surface integrity but also provide pathways for oxygen ingress, thereby facilitating the oxidation reaction. As the oxidation process advances, oxide accumulation gradually occurs, potentially leading to peeling off and contributing to material wear. This phenomenon underscores the intricate interplay between mechanical deformation, oxidation, and material degradation in the presence of aggressive ions like Cl^−^.

In PSR, the worn scar consisted of a central adhesive zone surrounded by a sliding zone, with the proportion of the adhesive zone increasing as the Cl^−^ concentration rises [45]. Clear cracks were visible on the adhesive surface, stemming from plastic deformation induced by stress concentration at the adhesive interface, as shown in Figure 10. However, compared to the morphology observed in GSR (as shown in Figure 5), the occurrence of cracks was significantly reduced. As evidenced b to Figure 3 and Figure 10, plowing grooves were observable on the worn surface, indicating the presence of abrasive wear. Furthermore, in GSR, debris particles formed by plastic deformation were readily expelled from the contact area by water flow, leading to the exfoliation of the oxide layer at the contact center. Conversely, in PSR, the discharge of wear debris was less efficient, resulting in the gradual accumulation of a third body layer in the central area over fretting cycles [18,46].

## 4. Conclusions

In this study, a comprehensive examination was conducted on the fretting wear behavior and damage mechanism in Inconel 600 alloy under different Cl^−^ concentrations. Through the analysis of experimental results, the following conclusions were drawn:

1. The variation in Cl^−^ concentration was found to exert a significant influence on fretting wear behaviors. As the Cl^−^ concentration increased, notable changes were observed in the fretting characteristics. Specifically, the onset time of friction coefficient fluctuation advanced progressively, indicating an earlier onset of wear-related instabilities. Concurrently, the wear volume exhibited a gradual increase with an increasing Cl^−^ concentration, suggesting a heightened propensity for material loss under more corrosive conditions.
2. In GSR, the influence of Cl^−^ concentration was notable. As the Cl^−^ concentration increased, the oxide layer on the worn surface tended to peel off, hindering the formation of a cohesive and protective oxide layer. Consequently, the wear mechanism transitioned gradually from abrasive wear to oxidation wear and delamination.
3. In PSR, the impact of Cl^−^ concentration on wear behavior was less pronounced compared to the GSR. The wear volume exhibited minimal sensitivity to changes in Cl^−^ concentration and remained notably lower than that observed in GSR. Moreover, the FC in PSR demonstrated relative stability without oscillatory behavior.

## Figures and Tables

**Table 1:** Chemical composition of Inconel 600 and 304SS (wt.%).

Specimen	Element									
	Cr	Fe	C	Si	Mn	P	Al	Ti	Co	Ni
Inconel 600	15.2	11.0	0.022	0.29	0.23	0.0038	0.23	0.30	0.06	Bal
	Cr	Ni	C	Si	Mn	P	S	Mo	Fe	
304SS	16.7	8.46	0.076	0.42	1.18	0.022	0.0082	0.18	Bal	

## Data Availability

Data is contained within the article.
